# Secondary Metabolites of *Hypericum leptophyllum* Hochst., an Endemic Turkish Species

**DOI:** 10.1100/2012/501027

**Published:** 2012-04-29

**Authors:** Necdet Camas, Jolita Radusiene, Zydrunas Stanius, Omer Caliskan, Cuneyt Cirak

**Affiliations:** ^1^Vocational High School of Bafra, Ondokuz Mayis University, Samsun, 55040 Bafra, Turkey; ^2^Nature Research Centre, Institute of Botany, Zaliuju ezeru 49, 08406 Vilnius, Lithuania

## Abstract

In the present study, the presence of the phloroglucinol derivative hyperforin, the naphthodianthrones hypericin and pseudohypericin, the phenylpropane chlorogenic acid and the flavonoids rutin, hyperoside, kaempferol, isoquercetine, quercitrine, and quercetine was investigated in *Hypericum leptophyllum* Hochst., an endemic Turkish species for the first time. The aerial parts representing a total of 30 individuals were collected at full flowering and dissected into floral, leaf, and stem tissues. After being dried at room temperature, the plant materials were assayed for secondary metabolite concentrations by HPLC. Aerial plant parts accumulated chlorogenic acid, hyperoside, isoquercetine, quercitrine, and quercetine, but they did not accumulate hyperforin, hypericin, pseudohypericin, rutin, and kaempferol. Accumulation levels of the detected compounds varied with plant tissues. Such kind of data could be useful for elucidation of the chemotaxonomical significance of the corresponding compounds and phytochemical evaluation of this endemic species.

## 1. Introduction 

The genus *Hypericum *L. from the family of *Hypericaceae* comprises more than 450 species divided in 36 sections with worldwide distribution in warm temperate, subtropical, and mountainous tropical regions [1]. Many herbs from genus *Hypericum* are pharmacologically important, particularly *Hypericum perforatum* L. which has been studied widely for its secondary metabolites composition and biological activity [2, 3]. Turkey is an important centre of *Hypericum *species origin and distribution where presently there are 89 registered species of which 43 are endemic [4]. All species of *Hypericum* from Turkish flora have been traditionally used as sedatives, antiseptics, and antispasmodics in folk medicine under the names: kantaron, peygamber çiçeği, kiliçotu, kanotu, kuzukıran, and binbirdelik otu [5]. *Hypericum leptophyllum* Hochst. which grows in dry stony or rocky calcareous zones of central Anatolia is one of the endemic species of Turkish flora. Plant stem is 20–60 cm in length, erect, or prostrate, branching from the base. Leaves are 5–35 mm in size, oblong, or linear to elliptic. Yellow flowers are numerous without black dots, like the leaves. Capsules are 5–10 mm in diameter with dorsal vital and lateral vesicles [4]. 

The major phytomedicinal compounds of *Hypericum* plants are thought to be phloroglucinol derivatives hyperforin and adhyperforin, naphthodianthrones hypericin and pseudohypericin, and the phenolics, as flavonoid hyperoside, rutin, quercitrin, quercetin and biapigenin, and phenylpropane caffeic and chlorogenic acids, which possess a wide array of biological properties [6]. Results from recent studies have indicated hyperforin as the main compound responsible for antidepressant effect of *Hypericum* extract, and it also exhibits anti-inflammatory and antiangiogenic effects [7]. The naturally occurring red pigments hypericin and pseudohypericin have been reported to exhibit important biological activities, namely, photodynamic, antiviral, antiretroviral, antibacterial, antipsoriatic, antidepressant, and antitumoral activities [8]. Flavonoids are a group of bioactive phenolics present in *Hypericum* plants. Results from clinical studies indicated the possible role of flavonoids in prevention of cardiovascular diseases and some kinds of cancer [9]. Although hyperforin and hypericins have been reported to mainly contribute to the pharmacological effects of *Hypericum* extracts, flavonoids have also made an important contribution to the antidepressant activity [10]. In this sense, many species of *Hypericum* from different localities of the world as well as Turkish flora have been investigated for the presence of the chemicals especially over the last decade [11–17]. However, to author's knowledge, there is no reported data on the chemistry of *H. leptophyllum*. The aim of the present study was to expand the knowledge on composition of flowering aerial parts of this endemic species in order to reveal the potential possibilities of using ones as pharmaceutical material. 

## 2. Material and Methods

### 2.1. Plant Material

The aerial parts of *H. leptophyllum *representing a total of 30 individuals were collected at full flowering in June of 2010 from Yozgat, Turkey (39° 50′ N Lat., 34° 48′ E Long., and 1420 m elevation). The plant material was dissected into floral, leaf, and stems tissues, dried at room temperature (20 ± 2°C). The species was identified by Professor Dr. Hasan Ozcelik, Faculty of Science and Art, Department of Biology, University of Suleyman Demirel, Isparta, Turkey. Voucher specimen was deposited in the herbarium of Vocational High School of Bafra (BMYO no. 127), Ondokuz Mayis University. 

### 2.2. Preparation of Plant Extracts

Air-dried plant material was mechanically ground with a laboratory mill to obtain a homogenous drug powder. Samples of about 0.5 g (weighed with 0.0001 g precision) were extracted in 50 mL of 100% methanol by ultrasonication at 40°C for 30 min. in a Sonorex Super model RK 225H ultrasonic bath. The prepared extracts were filtered through a membrane filter with pore size of 0.22 *μ*m (Carl Roth GmbH, Karlsruhe, Germany) and kept in a refrigerator until analysis no longer than 3 hours. The extracts for naphthodianthrones analysis after ultrasonication were exposed to light for 30 min. due to the photoconversion of protohypericin into hypericin and protopseudohypericin into pseudohypericin.

### 2.3. HPLC Analysis 

A Shimadzu Prominence LC-20A (Shimadzu Europa GmbH, Duisburg, Germany) chromatographic system equipped with two LC-20AD model pumps, a SIL-20AC auto-injector, a thermostat CTO-20AC, and a SPD-M20A detector was used for HPLC analysis. Separation of all compounds was carried out using an YMC Pack Pro-C18 (YMC Europe GmbH, Dinslaken, Germany) column (150 mm × 4 mm i.d.; 3 *μ*m particle sizes) with 10 mm guard precolumn. The mobile phase consists of solvent A (water containing 0.1% trifluoroacetic acid (TFA)) and solvent B (acetonitrile containing 0.1% TFA). The following binary gradient elution program was used: 0-1 min (B 5→5%), 1–14 min (B 5→20%), 14–20 min (B 20→80%), 20–30 min (B 80→100%), 30–39 min (B 100→100%), 39–39.5 min (B 100→5%), and 39.5–45 min (B 5–5%). The mobile phase was delivered with a flow rate of 1.0 mL min^−1^; volume of extract injected was 10 *μ*L. Detection was performed at 210–790 nm wave length range with a constant column temperature at 40°C. The eluted compounds were identified on the basis of their retention time by comparison with retention time of reference standards and also confirmed with UV spectra of reference standards in the wavelength range from 210 to 790 nm. 

**Figure 1:**
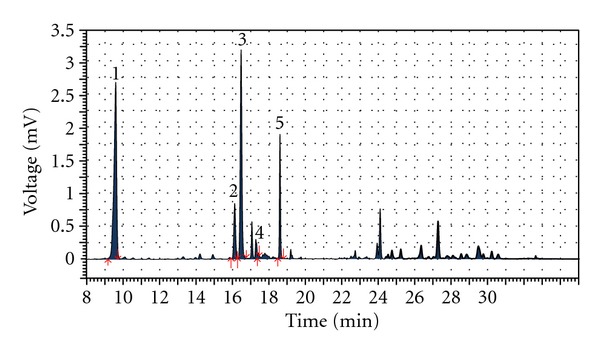
HPLC chromatogram of *Hypericum leptophyllum *methanolic extract detected by UV at 353 nm wave length. Peak identified: 1—chlorogenic acid (retation time (*t*_*R*_)—9.61 min.), 2—hyperoside (*t*_*R*_—16.13 min.), 3—isoquercetine (*t*_*R*_—16.49 min.), 4—quercetrine (*t*_*R*_—17.45 min.), 6–quercetine–(*t*_*R*_—18.62 min.).

The hypericin and pseudohypericin elution program was isocratic. The mobile phase consists of acetonitrile containing 0.1% TFA. Flow rate of mobile phase was 1.1 mL·min^−1^. Ten micro liters of extracts were injected. Detection was recorded at 210–790 nm wave length range with a constant column temperature at 40°C. The quantification of detected compounds was achieved by using external standard method at the maximal absorption on the UV spectra of corresponding compounds: chlorogenic acid—325 nm, rutin—353 nm, hyperoside—353 nm, isoquercetrine—353, kaempferol—346 nm, quercetrine—347 nm, quercetine–368 nm, hyperforin—270 nm, and hypericin and pseudohypericin—580 nm wavelength. A six-point calibration curves were obtained with pure standards dissolved in MeOH in the concentration range of 0.2–110 *μ*g/mL. All calibration cures showed good linear regression (*r* ^2^ > 0.999) within the test range. Typical HPLC chromatograms of *Hypericum leptophyllum *flower extract are shown in Figure 1. All solvents and standards of reference substances were of HPLC grade and purchased from Roth Chemical Company (Karlsruhe, Germany).

## 3. Results and Discussion

In the present study, we observed that the aerial plant parts accumulated the phenylpropane chlorogenic acid and the several flavonoids, namely, hyperoside, isoquercetine, quercitrine, and quercetine. We did not detect phloroglucinol derivative hyperforin and naphthodianthrones as hypericin and pseudohypericin. The accumulation of hypericins is produced in the dark glands, and the occurrence of dark glands in an organ is regarded as a reliable indicator of the presence of hypericins in a given species [1]. Morphologically we observed no dark glands on the aerial parts of *H. leptophyllum *plants, and this is a possible reason why plant tissues do not accumulate hypericins. 

Accumulation levels of the detected phenolic compounds varied with plant tissues. Isoquercetine, quercitrine, and quercetine were accumulated mainly in flowers, while leaves were found to be superior over flowers in terms of chlorogenic acid and hyperoside accumulations (Table 1). The differences in chemical composition between leaves and flowers found in the present study for the *H. leptophyllum *largely correspond to those described for *H. perforatum*, whose flowers accumulated larger amounts of quercetin and quercitrin and whose leaves had the highest level of hyperoside [19]. In other species of *Hypericum,* similarly, quercitrine and quercetine were accumulated mainly in floral parts while leaves produced higher amounts of chlorogenic acid and hyperoside in *H. origanifolium* [20], *H. perfoliatum *[21], and* H. maculatum* [18].

Results from the present study indicate that *H. leptophyllum *accumulates lower concentrations of quercetine and quercitrine, comparable concentration of hyperoside, and higher concentration of chlorogenic acid when compared to *H. perforatum*, a well-known and commercial source of the compounds examined (Table 2).

## 4. Conclusions

In conclusion, there is an increasing interest in recent years for using medicinal and aromatic plants as natural sources in pharmaceutical, food, biotechnology, agricultural, and cosmetic industries all over the world. Thus, efforts have been made to discover new sources of potential biological active secondary metabolites. In this sense, the present results encourage further studies on documenting potential pharmacological activity of *H. leptophyllum*. Here, the presence of bioactive compounds examined in this endemic plant was reported by us for the first time. Such kind of data could also be useful for elucidation of the chemotaxonomical significance of the corresponding compounds and phytochemical evaluation of* H. leptophyllum*.

## Figures and Tables

**Table 1:** Secondary metabolites content (mg/g DW) in stem, leaf, and flower of *Hypericum leptophyllum*.

Plant parts	Chlorogenic acid	Hyperoside	Isoquercetine	Quercitrine	Quercetine
Flower	17.47	2.69	16.98	0.10	2.39
Leaf	24.55	3.74	10.58	0.09	2.19
Stem	14.01	1.84	8.42	0.00	1.61

**Table 2:** Comparison of the content (mg/g DW) of chlorogenic acid, hyperoside, isoquercetine, quercitrine, and quercetine in *Hypericum leptophyllum *and *Hypericum perforatum *(compiled from referred sources).

Compounds	*H. leptophyllum*	*H. perforatum*	References
Chlorogenic acid	14.01–24.55	1.11–2.19	Maggi et al. [13]
Hyperoside	1.84–3.74	2.07–7.69	Maggi et al. [13]
Isoquercetine	8.42–16.98	—	No previous report
Quercitrine	0.00–0.10	0.05–4.77	Radusiene et al. [18]; Mártonfi et al. [15]
Quercetine	1.61–2.39	0.05–24.12	Radusiene et al. [18]; Mártonfi et al. [15]
